# Mental health problems of front-line medical staff in the early stage of public health emergencies

**DOI:** 10.3389/fpsyt.2024.1377815

**Published:** 2024-04-26

**Authors:** Dong-Bao Wang, Jin-Bo Jiang, Hai-Jun Zhang, Di Wu, Ya-Hong Zhang, Long-Biao Cui, Jian Zhang, Xiao-Hui Wang

**Affiliations:** ^1^ Department of Psychiatry, Xijing 986 Hospital, Fourth Military Medical University, Xi’an, China; ^2^ Department of Pulmonary and Critical Care Medicine, Xijing Hospital, Fourth Military Medical University, Xi’an, China; ^3^ Department of Psychiatry, 93307 Military Hospital, Shenyang, China; ^4^ Department of Psychiatry, Xijing Hospital, Fourth Military Medical University, Xi’an, China; ^5^ Department of Psychiatry, Xi’an Gaoxin Hospital, Xi’an, China; ^6^ Shaanxi Provincial Key Laboratory of Clinic Genetics, Fourth Military Medical University, Xi’an, China; ^7^ Department of Respiratory Medicine, Xi’an People’s Hospital (Xi’an No. 4 Hospital), Xi’an, China; ^8^ Department of Pulmonary and Critical Care Medicine, The First Affiliated Hospital of Chongqing Medical University, Chongqing, China

**Keywords:** public health, medical staff, mental health, influencing factors, coping strategy

## Abstract

In the face of the unprecedented public health crisis caused by the novel coronavirus pneumonia epidemic, front-line health workers are under enormous mental pressure. This paper aims to explore the mental health challenges faced by front-line health workers in the early stages of a public health emergency, such as stress, anxiety, and depression. At the same time, the factors that increase their mental stress are analyzed, and practical measures are put forward to prevent and manage mental health problems, aiming at improving the quality of medical treatment during public health emergencies. This paper has some reference value for people engaged in mental health prevention.

## Introduction

**Figure 1:**
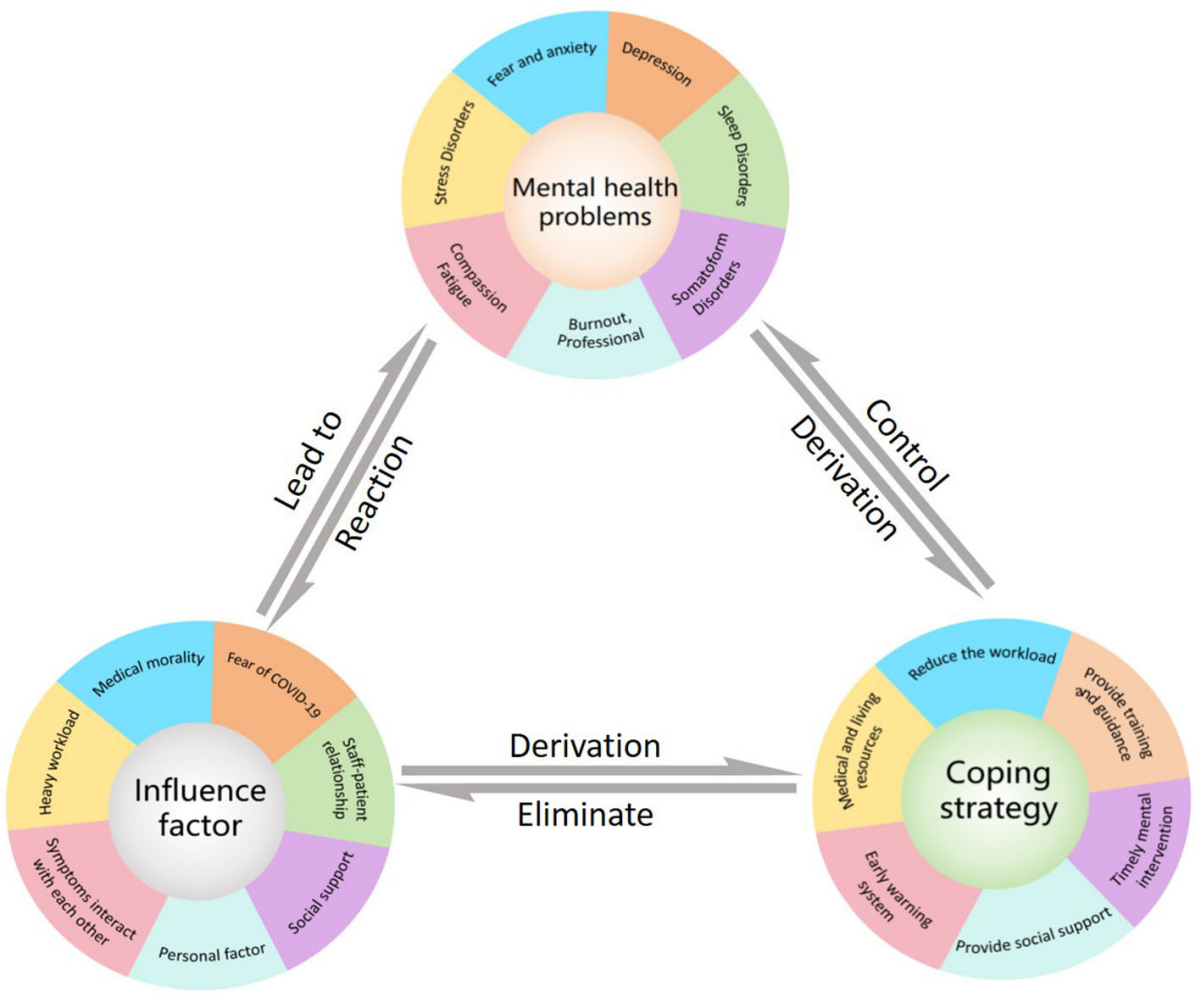
Summary of this article.

Public health emergencies refer to sudden and significant impacts on public health such as major infectious disease epidemics, mass unexplained diseases, and major food and occupational poisonings. These emergencies can cause or may cause serious damage to public health (1). COVID-19 emerges and spreads rapidly across the globe due to its high infectiousness, frequent mutations, and high mortality in late 2019. The World Health Organization (WHO) declared it a global pandemic in March 2020, and a significant public health emergency (2). The COVID-19 epidemic and the measures taken to prevent and control its spread have had a profound impact on people’s lives, resulting in mental health issues for some individuals. A survey of 1,210 people was conducted between January and February 2020. The survey found that 54% of respondents experienced moderate to severe impacts on their mental health due to COVID-19, with 29% experiencing moderate to severe anxiety symptoms and 17% experiencing moderate to severe depressive symptoms (3). The impact of mental health issues in public health emergencies cannot be ignored (4). Medical staff in front-line positions face infection risk and pressure to fight the epidemic, requiring special attention to their mental health status during the pandemic (5). During the COVID-19 pandemic, healthcare workers often experience mental health problems such as burnout, stress, anxiety, depression, sleep disorders, and post-traumatic stress disorder. A meta-study at the beginning of the pandemic assessed the overall prevalence of anxiety in front-line healthcare workers at 25.8%, depression at 24.3%, and stress at 45.0% (6). Another meta-analysis of front-line healthcare workers found that the prevalence of anxiety, depression, and sleep problems was 28.68%, 32.81%, and 50.86% respectively, compared with 25.91%, 26.01%, and 34.08% in Non-frontline areas (7). Currently, the most relevant studies are cross-sectional surveys of doctors and nurses using psychological questionnaires to assess the incidence of mental health problems and related risk factors. However, even in meta-analyses, results are not entirely consistent, which may be due to various factors such as sample representativeness, timing during the COVID-19 pandemic, geography, and vaccination policy. Importantly, there is currently no universal or unique incidence of mental health problems. However, numerous studies have shown that healthcare workers’ mental health significantly impacts public health events. The WHO has recently emphasized the need for preparedness in the fight against the X virus. Although the COVID-19 pandemic has ended, we should take this opportunity to review the mechanisms of emergence and impact, summarize interventions, and prepare for future public health emergencies facing unknown X virus. Attention to health workers’ mental health during an epidemic is therefore important not only for their own physical and mental health but also for treating patients and controlling public health events. **Figure 1** is a summary of the review.

## Methods

With “mental health”, “mental disorder”, “stress”, “anxiety”, “depression”, “sleep disorder”, “burnout”, “medical staff/doctor/nurse”, “COVID-19”, “impact”, “Factors”, “countermeasures”, and other subject words or keywords, we searched PubMed, Web of Science, Sino-Med, and CNKI literature databases, and then screened and sorted out the literature. Taking PubMed as an example, the search formula was as follows: (((COVID-19[MeSH Terms]) OR (COVID-19[Title/Abstract])) AND ((((((Stress, Psychological[MeSH Terms]) OR (Anxiety[MeSH Terms])) OR (Depression[MeSH Terms])) OR (Sleep Initiation and Maintenance Disorders[MeSH Terms])) OR (dyssomnias[MeSH Terms])) OR (((Mental Health[MeSH Terms]) OR (Mental Health[Title/Abstract])) OR (mental disorders[MeSH Terms])))) AND ((Medical Staff[Title/Abstract]) OR (Medical Staff[MeSH Terms])).

### Main symptoms

A meta-analysis of 292,230 participants from 46 countries by Zhu H et al. showed that the incidence of mental health problems among healthcare workers during the pandemic: 37.1% reported anxiety, 37.6% reported depression, 43.7% reported insomnia, 41.3% reported stress, 30.6% reported post-traumatic stress disorder, and 37.1% reported depression. 43.6% reported burnout and 25.0% reported somatic symptoms. Their study was comprehensive, and a subgroup analysis of frontline and non-frontline personnel was compared (8). **Table 1** shows the data we used. **Table 2** is a summary of the original studies.

**Table 1:** Incidence of common mental health problems among medical personnel (8).

Category	Overall prevalence% (95% CI)	Frontline prevalence% (95% CI)	Non-frontline prevalence% (95% CI)
Stress	41.3 (35.1~47.9)	47.0 (38.2~56.0)	31.4 (20.0~45.5)
Anxiety	37.1 (34.7~39.7)	41.4 (37.6~45.3)	27.6 (24.3~31.2)
Depression	37.6 (35.0~40.4)	41.8 (38.0~45.7)	30.7 (27.1~34.7)
Insomnia	43.7 (39.1~48.5)	49.5 (44.0~55.1)	29.9 (24.8~35.4)
PTSD	39.6 (29.6~50.6)	35.8 (24.4~49.1)	28.7 (20.5~38.7)
Burnout	43.6 (36.3~51.2)	42.0 (33.2~51.4)	36.1 (21.8~53.4)
Somatoform Disorders	24.4 (10.5~47.1)	30.7 (18.6~46.2)	27.7 (11.5~53.1)

Data in **Table 1** are from reference (8).

**Table 2:** Summary of the original studies included in the review.

Authors and Study time	The Sample included (n)	Instruments used	Main findings
Lu W, et al 2020.02 (9)	2042 doctors and nurses	Hamilton Anxiety Scale (HAMA) and Hamilton Depression Scale (HAMD)	70.6% reported moderate to severe fear, 25.5% anxiety, and 12.1% symptoms of depression
Liao R, et al 2020.02 (10)	1480 nurses	Toronto Alexithymia Scale (TAS-20) and Impact of Event Scale-Revised (IES-R)	Alexithymia Scale and Event Impact Scale scores (53.29 ± 13.74) and (25.38 ± 17.45)
Liu M, et al 2020.02 (11)	219 doctors and nurses	Professional Quality of Life Scale (ProQOL) and Connor-Davidson Resilience Scale (CD-RISC)	Moderate mild, moderate, and severe empathic fatigue 47.95%, 14.61%, 10.5%
Wang H-J, et al 2020.02 (12)	1045 doctors and nurses	Hospital Anxiety and Depression Scale (HADS), Perceived Stress Scale (PSS-14), and Insomnia Severity Index (ISI)	Anxiety, depression, insomnia, and stress levels were higher in the high-risk group than in the low-risk group
Zhai X-Y, et al 2020.02 (13)	1005 doctors and nurses	Generalized Anxiety Disorder (GAD-7), Patient Health Questionnaire (PHQ-9), and Patient Health Questionnaire (PHQ-15)	20.20% reported anxiety symptoms, 33.63% depression, and 37.41% somatization symptoms
Chen L-Q, et al 2020.03 (14)	828 Nurses	Self-designed questionnaire	73% reported panic and 55% anxiety
Zhuang L-L, et al 2020.03 (15)	1100 nurses	Professional Quality of Life Scale (ProQOL)	The empathic fatigue was higher than the norm
Jaison Jacob, et al 2020.07 (16)	803 doctors and nurses	Depression, anxiety, and stress scale (DASS-21) and screener for somatoform disorder (SSD)	35.4% reported symptoms of anxiety, 27.4% somatoform disorders, 23% depression, and 14% stress
CHU J-X, et al 2020.07 (17)	8316 doctors and nurses	GAD-7, PHQ-9, Athens Insomnia Scale (AIS), and impact of events scale- revised (IES-R)	51.54% reported anxiety symptoms, 55.54% depression, 67.02% physical problems, 46.75% insomnia problems, and 23.30% PTSD
Mei S-L, et al 2020.08 (18)	516 doctors and nurses	PTSD Checklist-Civilian Version, Perceived Stress Scale (PSS), and Insomnia Severity Index (ISI)	10.5% PTSD symptoms, and insomnia mediates the impact of stress perception on PTSD
Wang X, et al 2021.03 (19)	1582 nurses	Professional Quality of Life Scale and Chinese 10-item Connor-Davidson Resilience Scale	Moderate degree of sympathy satisfaction (36.99 ± 6.71), burnout (24.14 ± 5.33), and secondary traumatic stress (24.53 ± 5.24)
Liu Y-F, et al 2021.12 (20)	1795 doctors and nurses	PTSD Checklist-5 (PCL-5) and Center for Epidemiologic Studies Depression Scale (CES-D)	16.40% reported PTSD and 18.30% reported depression

### Stress

The Stress response is a non-specific reaction that occurs in individuals when their bodies are exposed to different stressors. This response includes two categories: physiological reactions and psychological reactions. Physiological reactions comprise sympathetic nerve excitement, increased secretion of pituitary and adrenocortical hormones, increased blood sugar, blood pressure, accelerated heart rate, and breathing. Psychological reactions to stress include emotional reactions, self-defense reactions, and coping reactions. When an individual displays inappropriate psychological and physiological responses to stress, it is known as a stress disorder. The two major stress disorders are acute stress disorder (ASD) and post-traumatic stress disorder (PTSD).

During the early stages of the COVID-19 pandemic, stress reactions were common among medical staff, with a detection rate of 73.4% in the early stages of the pandemic (21). A study found that 27.39% of medical staff suffered from acute stress disorder, with women reporting higher stress scores than men. In 2020, a survey of 1,480 nurses working on the frontlines revealed that the incidence rates of mild, moderate, and severe stress reactions were 31.62%, 26.15%, and 17.91%, respectively (10). As mentioned above, the prevalence of stress among hospital staff caring for COVID-19 patients was 45% (95% CI 23.4% to 67.5%) (6). The COVID-19 pandemic has significantly affected the mental health of healthcare workers, particularly female healthcare workers who are experiencing a higher incidence of stress disorders.

Health workers may develop post-traumatic stress disorder (PTSD) as a result of experiencing extreme stress during public health crises. PTSD is characterized by symptoms such as reliving the traumatic event, avoiding situations that remind them of the event, persistent emotional numbness, heightened alertness, and irritability. Healthcare workers on the front lines of battling diseases such as Severe Acute Respiratory Syndrome (SARS), Middle East Respiratory Syndrome (MERS), and Ebola in Africa are often exposed to a large number of deaths, which can have a significant impact on their mental well-being. A study of healthcare workers during the SARS epidemic found that 25.8% of subjects reported PTSD symptoms (22). In a study in August 2020, 10.5% of 516 healthcare workers between the ages of 21 and 65 years had PTSD symptoms (18). Another study conducted in July-August of the same year, involving 8,316 healthcare workers, found that PTSD was as high as 23.3% (17). A recent multicenter study found that 16.40% of health workers (295 out of 1,795) still had post-traumatic stress disorder (PTSD) two years after the outbreak. The study found that PTSD was correlated with factors such as participating in clinical work, working in an unsafe environment, and having a poor doctor-patient relationship (20).

### Fear and anxiety

Fear is a response to events that are threatening, which leads to certain behaviors that help us cope with or avoid these threats. In mild cases, this reaction may manifest as fear or apprehension, while in severe cases, it can result in panic and anxiety, accompanied by symptoms such as palpitations, shortness of breath, trembling limbs, and excessive sweating. Fear can cause long-lasting anxiety, even after the danger has passed. Pathological anxiety is a persistent feeling of tension, worry, and fear without a rational basis. It’s frequently linked to physical and autonomic dysfunctions observed in anxiety disorders.

The 2020 Journal of Anxiety Disorders Editor’s Note titled ‘Coronavirus Phobia: Fear and the 2019-nCoV Epidemic’ proposes that the anxiety surrounding COVID-19 is due to its sudden emergence and the ambiguity surrounding the severity of the outbreak at the time. The note highlights that the fear of COVID-19 is much greater than the fear of seasonal flu, despite the latter causing more fatalities (23). A survey conducted in May 2020 found that hospital staff who had no direct contact with COVID-19 patients reported lower levels of fear than frontline healthcare workers (24). At the onset of the epidemic, 73% of nurses in the sentinel hospitals for fever patients were afraid, with 13% being very afraid and only 21% not being afraid (14). In April 2020, a meta-analysis revealed that up to 67% of medical staff experience anxiety-related symptoms (25). Medical staff experienced more fear (70.6%) than administrative staff (58.4%). Additionally, high-risk exposure employees had a significantly higher prevalence of fear and were 1.4 times more likely to experience it than non-clinical exposure staff. The fear experienced by the staff was due to psychological stress from isolation, infection fear, lack of protective equipment, outbreak uncertainty, dissatisfaction with work, and loneliness (9).

Individuals who are at risk of contracting diseases, including epidemics, and those who have concerns regarding their personal and family health, may experience severe symptoms of anxiety. In a survey conducted among healthcare workers in sentinel hospitals in February 2020, it was found that anxiety was prevalent among 23.04% of the participants. The rates of mild, moderate, and severe anxiety were 16.09%, 16.09%, and 2.17%, respectively. The study also revealed that anxiety was more common in females than males (25.67% vs. 11.63%) and in nurses compared to physicians (26.88% vs. 14.29%) (26). The severity of anxiety symptoms among healthcare workers during COVID-19 was also related to specialty and level of exposure. One meta-study found that 27% of healthcare workers had anxiety symptoms, with a subgroup analysis of the overall prevalence of anxiety of 35.21% for frontline staff and 21.15% for non-primary medical staff (7). A February 2020 survey found that 22.6% of medical staff had mild to moderate anxiety, 2.9% had severe anxiety, and the corresponding proportions for administrative staff were 17.1% and 2.9%, respectively. The prevalence of anxiety was significantly higher in high-risk contact departments compared with non-clinical and low-risk contact departments. Respiratory, emergency, intensive care, and infectious disease nurses had twice as much anxiety and depression as non-exposed nurses (9). Unresolved psychological conflicts can cause symptoms of anxiety, which may hinder healthcare workers’ performance (6).

### Depression

The primary symptoms of a depressive episode include depressed mood, decreased interest, and anhedonia. They are often associated with anxiety, cognitive changes, somatic symptoms, and behavioral symptoms, including inattentiveness, unresponsiveness, sleep disturbance, reduced volitional and behavioral activity, and tiredness. A February 2020 study found that the incidence of depression was significantly higher among healthcare workers in the high-risk group than those in the low-risk group (43.6% vs 36.8% p=0.028). Moderate to severe depressive symptoms were also more common in the physician group (12). A meta-analysis of frontline healthcare workers found that 29% of them exhibited depressive symptoms (7). Meanwhile, a separate meta-analysis found that the prevalence of depression among health workers in the designated hospitals during the COVID-19 outbreak (December 2019 - April 2021) was 31.0% [95% CI (0.25, 0.37)]. Subgroup analysis showed a higher prevalence of depression among women, married, bachelor’s degree or higher, nurses, and junior/non-frontline health workers (27). Two years after the outbreak, 329 out of 1795 (18.30%) health workers were still experiencing depressive symptoms. Clinical involvement, job insecurity, poor doctor-patient relationships, poor health, job dissatisfaction, and low family support were associated with a higher risk of depression (20). The New Crown outbreak’s impact on health workers continues, and their mental health deserves continued attention in a catastrophic health emergency like COVID-19.

### Sleep disorders

Sleep disorders develop over time as innate sleep cycles and patterns are disrupted by trauma, stress and life events, personality, emotional state, and environment. Stressful work environments and workloads in designated hospitals can disrupt healthcare workers’ sleep patterns. This can lead to insomnia, disturbed sleep patterns, and nightmares. Chronic sleep deprivation in healthcare workers can lead to physical fatigue, low energy, and poor appetite the next day, as well as impairing cognitive function, reducing attention, and increasing the likelihood of errors. A cross-sectional survey of 543 frontline healthcare workers was conducted in February and March 2020. The study found that 216 (39.8%) of the respondents reported sleep problems. Anxiety and depressive symptoms were also associated with difficulty sleeping (28). A meta-analysis conducted on 17 April 2020 estimated the prevalence of insomnia among healthcare workers during the epidemic to be 38.9% (29). Another meta-analysis the following year showed a 40% prevalence of insomnia among healthcare workers during the epidemic (7). A further meta-analysis of frontline workers showed that the prevalence of sleep disorders was 34.8% (95% CI: 24.8 - 46.4%) in nurses and 41.6% (95% CI: 27.7 - 57%) in doctors (30).

### Somatoform disorders

Somatoform disorders involve physical symptoms that have no apparent medical cause. These conditions arise due to emotional distress and may include pain, numbness, paralysis, blindness, hearing loss, or other physical problems. The affected individuals may use these physical symptoms to express or relieve their emotional problems. A February-March 2000 survey using the PHQ-15 found that 37.41% (376 of 1007) of hospital health workers had physical symptoms (13). In June-July of the same year, a study reported 27.4% physical symptoms among healthcare workers, with nurses (28.8%) having a significantly higher prevalence than doctors (18.2%) (16). The study found that medical staff working in square pod hospitals had a higher incidence of somatization than those working in general hospitals, and there was no significant reduction in somatization symptoms on return to their local hospitals (31). It is important to note that some medical staff may have symptoms such as somatoform disorders or sleep disorders rather than obvious mental health problems such as anxiety and depression.

### Burnout

Burnout is a psychological syndrome. It results from long-term work pressure that is not effectively managed. It is characterized by emotional exhaustion, dehumanization, and low personal accomplishment. In 2019, the WHO officially classified burnout as a syndrome in the 11th revision of the International Classification of Diseases (ICD-11). Front-line healthcare workers may experience burnout due to long working hours, inadequate resources, lack of control over their work, and adverse working conditions. An overall prevalence of 43.6% (95% CI: 36.3% - 51.2%) was found in the meta-analysis of burnout among health workers during the COVID-19 pandemic (8). Importantly, the COVID-19 pandemic resulted in a significant increase in workload for healthcare workers in respiratory, intensive care, and infectious disease units. The prevalence of burnout among these workers has raised concerns. A meta-analysis of ICU physicians and nurses found that a significant number reported high job boredom. Specifically, 41% of 8,187 ICU doctors and 44% of 12,536 nurses reported high levels of boredom. The study also found that the prevalence of occupational boredom among ICU nurses was higher than usual during the epidemic (0.61 vs 0.37). Furthermore, the proportion of ICU nurses with high emotional exhaustion was higher than physicians (0.42 vs 0.28) (32). Another study found that respiratory physicians and therapists had a significantly higher than usual prevalence of professional boredom during the epidemic (68.4% vs 41.6%), and a significant association between COVID-19 coverage and incidence (33). Respiratory, infectious, critical care and other specialists’ work is closely linked to COVID-19 infection and requires sustained attention and proactive interventions to reduce burnout risk.

### Compassion fatigue

Compassion fatigue is common among medical and relief workers, who are often exposed to negative emotions like pain and grief for long periods during rescue, which can lead to psychological stress like apathy. It is characterized by fluctuating feelings, reduced empathy, stereotyped and impressionistic treatment of patients, and symptoms of irritability. It includes a combination of two measures: burnout (chronic occupational stress that reduces willingness to work) and secondary trauma (traumatic symptoms arising from prolonged exposure to the suffering of others). Healthcare workers had low sympathy satisfaction during the COVID-19 pandemic. There may also be an increase in the likelihood of workers leaving front-line roles, such as intensive care units. An online survey of over 1,500 frontline nurses between 9-15 March 2020 found that these nurses experienced moderate compassion satisfaction (36.99 ± 6.71) and burnout (24.14 ± 5.33) (19). In the same year, another researcher surveying health workers found that the prevalence of mild, moderate, and severe compassion fatigue was 47.95%, 14.61%, and 10.50% respectively (11). A recent study found that 91.7% of nurses experienced low or moderate compassion satisfaction in over 1,100 nurses in Designated hospitals. The compassion satisfaction survey score was 32.63 ± 6.46. The study also concluded that compassion fatigue is associated with several factors. These include workplace, exposure history, loneliness, work environment, sleep, and resilience (15).

### What causes

Research has shown that the mental well-being of frontline health workers during an epidemic is influenced by objective factors such as work pressure, infection, epidemic uncertainty, prolonged social isolation, and inadequate psychological support (5, 9, 34, 35). In addition, personal circumstances, including gender, age, and psychological resilience, may also have an impact. **Table 3** shows a summary of the influencing factors.

**Table 3:** Summary of factors affecting common mental health problems.

Category	Detail list
Workload	Long working hours, heavy workload, and frequent duty
Fear of the COVID-19	Fear of being infected with the virus and infecting others
Ethical consideration	Blame for patient deaths and unequal distribution of medical resources
Social support	Lack of communication with family members and friends
Personal factor	Gender, age, marriage, and personality

### Workload and working hours

High levels of work-related stress can have a detrimental impact on the motivation, mental health, and medical behavior of healthcare workers. Therefore, it is crucial to identify the causes and severity of work stress among frontline health workers during epidemics. This will help protect and safeguard healthcare workers while also improving the quality of patient care (6). At the beginning of the epidemic, frontline healthcare workers faced an immense burden. They had to deal with numerous cases, providing treatment to severely ill patients, and implementing complex interventions. A study revealed that workload and time allocation were the second most significant sources of work stress among frontline health workers during the epidemic (36). During that period, the challenging medical tasks and the shortage of medical and nursing care resulted in extended working hours. This, in turn, prevented medical and nursing staff from getting sufficient rest, leading to physical and mental exhaustion. Surveys conducted on frontline nurses showed a direct relationship between the number of hours they worked per day and their mental workload (37). Another study found that nurses who worked more night shifts reported higher levels of anxiety and were more likely to experience negative emotional reactions (38). Medical staff may experience feelings of loneliness and helplessness due to the long hours of intense work in a challenging environment. Furthermore, their mental well-being can be negatively affected by the constant need to learn and adapt to updated treatment guidelines and operating procedures (39).

### Ethical consideration

At the beginning of the pandemic, there were no effective drugs or established treatment options for novel coronavirus infection. This can create immense pressure on healthcare workers who witness patients deteriorating or dying. As a result, healthcare workers may experience feelings of frustration, powerlessness, self-blame, or guilt, which can harm their mental state (40, 41). A study of frontline health workers in designated supported this idea, finding that the presence or absence of experiencing death in patients with no coronary pneumonia was one of the main influences on vicarious traumatization (42). During the early stages of the epidemic, healthcare workers were faced with the overwhelming task of making critical medical decisions due to limited resources. These decisions had to be made quickly to reduce overall mortality and morbidity. Clinical decisions regarding the allocation and utilization of limited medical resources may differ from those made in standard medical practice. This can lead to various negative emotions such as helplessness, internal conflict, anxiety, depression, and compassion fatigue. It can also result in mental health problems such as PTSD (39, 43). Individuals may experience feelings of helplessness and anxiety, which can lead to mental health problems such as anxiety, depression, compassion fatigue, and post-traumatic stress disorder.

### Fear of the COVID-19

The original strain of the novel coronavirus is extremely virulent and easily transmissible. Frontline workers are worried not only about catching the virus themselves but also about the possible harm their diagnosis could have on their coworkers, family, and friends. This fear of infection only amplifies their psychological distress (39). Healthcare workers who are unable to provide personal care to a family member with a confirmed diagnosis may experience negative emotions such as guilt, loss, and helplessness. A study has shown that healthcare workers who have a family member, friend, or colleague diagnosed with an illness have higher overall vicarious traumatization scores (B=-9.707). Conversely, when healthcare workers encounter infected colleagues and experience emotions such as fear, helplessness, and worry, they also experience higher levels of psychological distress (42). A recent study discovered that healthcare workers on the frontlines of the outbreak experienced varying degrees of worry and stress. The age group of 31-40 expressed greater concern about their family members contracting the virus, as compared to other age groups. On the other hand, healthcare workers who were over 50 years of age were more stressed about the patients they were treating and their deaths. Lastly, the age group of 41-50 found that worrying about their safety was a significant factor that contributed to their anxiety levels. Studies conducted in workplaces have shown that as the likelihood and severity of exposure to novel coronaviruses decrease, frontline healthcare workers experience a reduction in anxiety, depression, and sleep disturbances (44).

### Lack of support

Prolonged isolation of frontline staff can lead to an increase in mental health problems such as anxiety and depression. This can be made worse by a lack of direct communication with family and friends, and a lack of emotional support and comfort, which can lead to increased feelings of isolation and powerlessness. Research has shown that positive support from family and loved ones can help reduce the psychological distress experienced by healthcare workers (38). Having high levels of family support can aid healthcare workers in adjusting to the challenging demands of their profession. On the other hand, low levels of family support are significantly linked to an increased risk and severity of PTSD and depressive symptoms. Apart from family support, getting support from colleagues and organizations at all levels can also act as a protective factor against PTSD in healthcare workers. This support serves as a buffer against the development of psychiatric disorders (20). Providing appropriate social support to health workers during an epidemic can reduce stress and anxiety and increase self-efficacy, thus improving their mental health (35).

### Personal factor

The mental health of healthcare workers can be affected by their circumstances and psychological resilience. A study found that female participants and nurses reported higher rates of affective symptoms compared to their male and physician counterparts, respectively (29). Female and nursing staff experienced higher levels of depressive symptoms than male doctors and other professionals, according to a survey of 1,156 medical staff. The study revealed that a higher percentage of women reported mild and moderate to severe anxiety (22.91%, 2.61%) than men (17.35%, 1.03%). Moreover, women experienced poor quality of sleep more frequently than men. He X-R et al. conducted a decision tree analysis study of over 15,000 medical workers and found that the detection rate of psychological abnormalities was higher in medical workers aged 30 to under 40 than in other age groups (45). As mentioned earlier, healthcare workers in the 31-40 age group are more concerned about household infections than other age groups (46). Other studies have found that the burnout rate of front-line personnel is significantly higher in the age group of 27-31 and 32-39, and the lowest in the age group of 40 and above (47). According to recent a study, healthcare workers’ job stress is influenced by various factors, including their previous experience with epidemics and their physical health. Caregivers who had prior experience with such outbreaks and were in good physical health reported lower total job stress scores. Additionally, family-related factors were found to be linked to higher levels of work stress for nurses who have two or more children, compared to their counterparts who have fewer or no children (36). A meta-analysis also confirmed that female healthcare workers who are married, have higher qualifications, hold entry-level positions, and work on the front line have higher rates of depression and anxiety compared to their peers (27). Personal factors such as gender, health status, family circumstances, and experience have to be taken into account when selecting front-line medical and nursing personnel for deployment in epidemic situations.

### How to do

The development of psychiatric symptoms into mental illness is a complex and dynamic process involving the interaction of several factors. An increased risk of underlying mental illness may be indicated by persistent and gradually worsening psychiatric symptoms. It may be a sign of developing mental illness when mental symptoms lead to impairment in a person’s daily life, work, and social functioning. Negative life events or psychological trauma, such as severe stress, unemployment, or family problems, can exacerbate mental symptoms and contribute to developing mental illness. Importantly, progression from mental symptoms to mental illness is not inevitable, and appropriate intervention or treatment can alleviate and manage symptoms in many people. Therefore, for healthcare workers with mental symptoms, early intervention and treatment are very important to prevent mental problems from further deteriorating into mental diseases. **Table 4** shows a summary of the response strategies.

**Table 4:** Summary of main coping strategies.

Category	Detail list
Workload	Adequate medical resources and medical staff, scientific work, and duty arrangements
Mental health training	Emergency plan, professional ability training, psychological education, and personalized guidance
Psychiatric intervention	Psychological support network, first-line psychiatrist consultation, and remote psychological services
Social support	Recognition awards, financial compensation, and care for family members
Working conditions	Improving working and living conditions and providing a rich and reasonable diet
Early-warning system	Early identification, regular investigation, drug treatment, and individualized psychological guidance

### Improving the working environment and system

The above information suggests that the heavy workload and long working hours harm the mental well-being of frontline health workers. It is therefore essential to improve the working conditions of frontline health workers. To address the shortage of medical staff in various departments, it is recommended to provide adequate medical resources and housing, which will help reduce workloads and distribute tasks evenly among individuals. Providing adequate medical resources and accommodation, which will help to reduce workloads and distribute tasks evenly among individuals, is recommended to address the shortage of medical staff in different departments. These measures can help alleviate negative emotions and promote a more positive mood. To establish an effective personnel deployment system, it is important to consider the physical condition and family situation of medical and nursing staff. The duty work system for doctors on duty, nurses on night shift, and emergency shifts should be arranged scientifically and reasonably. It is also necessary to coordinate the allocation of front-line positions and consider the specifics of working hours, rest, and isolation frequency (36). Conduct a detailed assessment to determine staffing needs in various departments and positions, and distribute the number of patients and medical/nursing staff reasonably and fairly among designated receiving facilities. Establish emergency medical reserve teams when needed to fill gaps in the team structure and to avoid backlog and shortage of medical and nursing staff.

### Mental health education and training

Health workers facing immense work pressure and mental stress during sudden epidemic disasters need specific mental health training to maintain their well-being. Leaders and organizers need to consider the whole situation and recognize the importance and key role of psychological support in health emergencies. An emergency mental health prevention and treatment policy for medical and nursing staff should be formulated as soon as possible, and a mental health team established, as well as specific measures for scientific and appropriate mental health education. Before frontline work, health workers should receive mental health training to improve their adaptability in emergencies and their ability to recognize and manage mental health problems that may arise during their work, including common professional knowledge about emergencies, mental health, and self-regulation. Mental health training provided during the epidemic should not be temporary, but a long-term and continuous support mechanism should be established. Provide regular online and offline training for staff on the various sources of stress they face in their work, as well as individual health counseling and emotional release training for staff in various contexts (37, 42, 48). Mental health training can improve medical staff’s ability to identify disasters, adapt positively, and regulate their emotions.

### Timely psychological interventions

To prevent psychiatric symptoms from developing into mental ill health, positive interventions and guidance should be offered to respond to the psychological problems of frontline health workers to avoid adverse effects on individuals and the team. To assist healthcare workers experiencing psychological discomfort during emergencies and crisis events, it is recommended that psychological support groups be established in various departments. These groups can provide emergency assistance and crisis intervention to help workers remain calm and capable of coping. Mental health professionals are deployed to provide psychological intervention and support to key front-line personnel. They offer both formal and informal support to help individuals cope with stress and improve their mental well-being. For the sake of accessibility and privacy, psychological support is available via the Internet and telephone, which enables health professionals to assess mental health, offer counselling, and provide early intervention (5, 49, 50). Developing supportive peer networks among healthcare workers can provide timely emotional support, coping strategies, and sharing of experiences to help build positive emotional and social support among one another. Improving psychological support and coping strategies during an emergency can help alleviate mental health problems among health professionals.

### Provision of social support

Outbreaks are significant public health events, and healthcare workers assume not only the personal role of physicians but also the key social role in public practice, requiring extensive societal and family support. Providing appropriate protective equipment and medical supplies can effectively alleviate staff concerns about infection risk and the psychological stress of treating patients. Recognition and appreciation of health professionals through social media and public opinion can positively impact their confidence and motivation to work. In addition, providing health workers with appropriate financial compensation and recognition, including allowances, subsidies, incentives, promotions, and recognition awards, can effectively reduce their financial burden during the epidemic, motivate them to remain in their positions, and reduce the stress and negative emotions caused by the epidemic. The authorities should ensure appropriate care and support for the families of health professionals, including immediate hospitalization of those with a diagnosis, timely support for families under quarantine due to the outbreak, and effective communication channels between health professionals and their families to address their concerns.

### Establishment of an early warning system

During the epidemic, it is important to promptly establish a mental health monitoring and assessment mechanism, which will allow regular monitoring of the mental health of frontline health workers, early identification of those suffering from mental health problems, and implementation of active interventions. By using questionnaires, psychological interviews, and telephone interviews, we can provide continuous mental health support and assessment to individuals who have experienced the death of a patient, infections of family members and coworkers, or other stressful events. This can help to manage negative emotions and alleviate psychological pressure. Healthcare workers receive regular psychological training to improve their ability to recognize the early signs of mental health problems in themselves and their colleagues. In the later stages of the epidemic, questionnaires, networks, or telephone calls can be used to track and follow up on the mental status of frontline health workers. This will help to understand the regression of symptoms such as depression and post-traumatic stress disorder and provide a basis for follow-up intervention and treatment.

## Discussion

Although the New Crown Pandemic has been declared over, this does not mean that our study is not relevant. Mental health problems play a very important role in public events. They affect the lives of individuals, the behavior of groups, and even the policies and actions of those in authority. We reviewed and summarized common mental health problems among frontline health workers during the COVID-19 outbreak, analyzed the main determinants of mental health problems, and proposed specific interventions. The review study provides several reference points for future public health emergencies. There have been many studies of the psychological impact of pandemic influenza on healthcare workers, but most have focused on the prevalence of psychiatric symptoms. Many original studies used different survey methods and psychometrics, which can bias results. In addition, the samples collected were affected by time, geographical, and cultural differences, the isolation and treatment measures used differed between countries, and the specific circumstances faced by healthcare workers were inconsistent. To provide more accurate data for local mental health prevention and control in the future, we need more targeted studies, for example on the mental health of healthcare workers in Asia. Second, as the pathogenicity of novel coronavirus variants decreases and outbreak prevention and control measures improve, the mental health status of healthcare workers will inevitably change dynamically in the face of the different phases of a new coronavirus pandemic, which is still poorly understood.

It Is worth noting that critical incident health worker mental health issues that may individually or collectively affect their mental health, such as stress, anxiety, and depression, are not isolated and may themselves be interrelated. One study showed that insomnia severity mediated the effect of perceived stress on PTSD among front-line health workers and that compassion fatigue moderated the perceived stress-PTSD relationship (18). Studies have found that circadian rhythm disturbances are associated with various mood disorders, and patients with depression have irregular biological rhythms in terms of sleep and cortisol levels, suggesting that circadian rhythms play a crucial role in the etiology and pathophysiology of depression (51). A previous study by our group also found an association between anxiety, stress, and sleep quality in COVID-19 field medical staff (52). The hormone cortisol can affect the human body in a variety of ways, including participating in stress responses, regulating anxiety levels, and affecting sleep quality. Some scholars have found that stress can cause the hypothalamic-pituitary-adrenal axis (HPA) cascade, resulting in the normal rapid feedback activation of glucocorticoid receptors/mineralocorticoid receptors expression downstream of astrocytes, resulting in short-term insomnia (53). Stress-induced changes in HPA axis activity are associated not only with acute insomnia but also with the maintenance of acute insomnia and the subsequent progression of insomnia disorder (54). Other studies have found that both anxiety and depression disorders may affect cortisol-mediated acute stress response and recovery secreted by HPA, that anxiety symptoms are associated with sluggish response to and recovery from acute psychosocial stressors, and that depressive symptoms are associated with steeper response and recovery slopes (55). In recent years, another quantifiable method of psychological assessment of stress and depression has been the use of cortisol and stress hormone measurements. Research has shown that measuring stress hormones is important for characterizing stress in the human body and correlating it with declining mental health. Therefore, the monitoring of specific biomarkers can be an aid in the early detection and diagnosis of psychiatric disorders (56).

In recent years, magnetic resonance imaging (MRI) has become a powerful tool for psychopathology, with multimodal MRI enabling qualitative and quantitative assessment of brain tissue at structural, functional, and molecular levels. This allows a deeper understanding of the mechanisms of psychiatric disorders and helps to translate psychopathology into clinical practice (57). It has been shown that morphological changes in the suprachiasmatic nucleus of the anterior hypothalamus and altered rhythms of light reception can lead to circadian rhythms influenced by rhythmic disruptions in HPA axis hormone release or monoamine neurotransmitter activity. Together with reduced melatonin release, reduced hippocampal neurogenesis, and disrupted striatal dopamine rhythms resulting from light phase disruption, these disrupted circadian processes play a key role in the development of depressive symptoms (51). It also found that individual differences in threat-related amygdala responses (assessed by functional MRI) predicted psychological vulnerability to life stress 1-4 years later and that this neuro biomarker predicted greater symptoms only in the context of relatively high life stress (58). Another study suggests that functional brain connectomes could be used to predict individual susceptibility to vicarious trauma, or as a neuromarker to identify people at high risk of depression or anxiety, by finding that pre-pandemic functional brain connectome measures predicted the level of vicarious trauma experienced by individuals at the peak of the pandemic (59). Further research, including appropriate animal models, combining molecular biology with functional imaging of human brains, could identify changes in internal structure and function of brain circuitry, and how specific changes in their timing affect mood. This will lead to a better understanding of the mechanisms by which mental disorders arise. It will be important for the development of intervention strategies, including new ones, for better prognosis and early treatment.

We can see that most of the research on the mental health problems of medical staff during the epidemic has been carried out using psychological questionnaires. Clinicians can understand a patient’s mental state, emotional problems, and behavior by asking them questions. Psychological questionnaires can suffer from issues with subjectivity and respondent inauthenticity. Therefore, they should be used in combination with other detection methods to improve accuracy and reliability. Biological detection techniques can help clinicians understand a patient’s physiology and thus better diagnose mental health problems. These include endocrine tests such as cortisol. By giving clinicians a complete picture of a patient’s mental state, neuroimaging techniques such as functional MRI and electroencephalography can provide a more accurate basis for diagnosing and treating psychiatric disorders. Comprehensive assessment using psychological questionnaires, biological tests, and neuroimaging will become more common. By integrating information from different sources, clinicians can make more personalized and accurate diagnoses and treatments, helping public health emergency workers to better manage mental health problems and improve the quality of their work.

## Author contributions

D-BW: Writing – original draft, Writing – review & editing, Data curation. J-BJ: Writing – original draft, Writing – review & editing, Data curation. H-JZ: Methodology, Writing – review & editing. DW: Methodology, Writing – review & editing. Y-HZ: Writing – review & editing. L-BC: Conceptualization, Supervision, Funding acquisition, Writing – review & editing. JZ: Conceptualization, Supervision, Writing – review & editing. X-HW: Conceptualization, Supervision, Writing – review & editing.
